# Loss of Time in the Treatment Adherence Process: A Qualitative Study in a Sample of Iranian People with Diabetes

**DOI:** 10.25122/jml-2019-0110

**Published:** 2020

**Authors:** Seyedeh Narjes Mousavizadeh, Zahra Banazadeh

**Affiliations:** 1. Department of Psychiatric Nursing, Nursing and Midwifery School, Shahid Beheshti University of Medical Sciences, Tehran, Iran; 2. Iran University of Medical Sciences, Tehran, Iran

**Keywords:** Treatment adherence, type 2 diabetes, qualitative study, grounded theory

## Abstract

Diabetes is a significant public health problem and one of the causes of death and disability globally. One of the main problems with diabetes control is the lack of adherence to therapeutic regimens in people with diabetes. This study investigates the experiences and views of the Iranian people with diabetes to identify the challenges of the process of adherence to treatment.

A grounded theory research design was used, incorporating in-depth interviews to collect the data. Using purposeful sampling, 28 people with type 2 diabetes (9 men, 19 women) from different places were included in the study. Constant comparative analysis was undertaken to identify key categories.

The main challenge in this process is losing the golden time of preventing the complications of the disease that occurs for the following reasons: cultural habits and values, religious beliefs (believing diabetes was God’s will), resistance to change due to age, job conditions, lack of harmony in the family, and non-shared decision-making in the health system.

People with diabetes go through trial and error in order to achieve awareness and insight, and consequently, adherence to treatment. Therefore, they need help and support to achieve insight and adherence to treatment faster and without complications. In fact, if the care plan is designed to encourage active patient participation by the treatment team in the shortest possible time, the time to achieve compliance will be shorter and will have the least side effects for these people.

## Introduction

Diabetes is a significant public health problem and a leading cause of death and disability worldwide [1]. The World Health Organization reported that the total burden of deaths from high blood glucose in 2012 had been estimated to 3.7 million [2]; it is predicted that this figure will double by 2030 [3]. In addition, diabetes is a costly disease due to its complications [4-6]. Prevention of complications and successful control of diabetes requires preventive and therapeutic practices. This is only possible with the active participation of patients in treatment and adherence to recommendations of the health providers; this practice is called adherence to treatment [7]. 

Studies have shown that adherence to treatment is the most important behavior related to diabetes management, which guarantees health or reduces the severity of symptoms [8, 9]. However, adherence to treatment in these people is relatively low, and numerous studies have been conducted in Iran has reported poor diabetes control [10-13]. According to the World Health Organization, the average follow-up treatment of people with chronic diseases in developed countries is reported to be 50%, but this rate is lower in developing countries [14]. Failure to adhere to the recommended treatment is one of the reasons for treatment failure, increased complications, increased length of treatment, and the rising costs of health care [15]. Many factors are involved in the failure to adhere to treatment, and the health care team and health care system planners must recognize these factors, as effective management can reduce the severity of disease complications [16].

Using a quantitative approach, several studies have investigated the factors involved in following the treatment of Iranian patients with diabetes [17-21], but despite the importance of the phenomenon of compliance, the treatment regimen and the severe consequences of non-compliance, such as the impact on the physical, mental, economic, social and familial status have not received much attention so far in a comprehensive study in Iran. Therefore, the current study was conducted to investigate the perspective and experience of Iranian people with type 2 diabetes to identify their needs, preferences, abilities and understanding, social context and all factors influencing treatment adherence.

## Material and Methods

This study was a qualitative, grounded theory study. The qualitative research method using the grounded theory approach was appropriately adapted to allow researchers to answer the central question of this study (how adherence to treatment occurs in people with type 2 diabetes? what are the factors influencing this process?) by using real experiences, free expression of feelings and behaviors [22, 23] in people with type 2 diabetes. For this purpose, the target-based sampling method (purposeful sampling) was used with maximum diversity [24]. It involves selecting participants who have the first-hand experience of the social process or phenomenon and provide the researcher with the necessary information about the phenomenon under study [25]. Considering the aim of the study, Iranian people with type 2 diabetes who met the inclusion criteria were selected as the main participants. The inclusion criteria for the study were: being Iranian, speaking Persian, having the diagnosis for more than a year, having the ability and willingness to express experiences of following a diet therapy in diabetes, not suffering from mental or cognitive disorders and incurable diseases such as cancer or the acquired immune deficiency syndrome. Maximum diversity in terms of age, sex, duration of illness, family history of diabetes, level of education, place of residence (city or village), and type of treatment was followed in the selection of participants. Sampling continued from December 2017 to July 2018 until data saturation was achieved, and during this study, 28 people with type 2 diabetes have entered the study. The exclusion criteria for the study were death or critical conditions. Each participant who lacked such conditions completed the study.

Data were collected using in-depth unstructured interviews. Each interview lasted between 40 and 120 minutes, and the participants were interviewed one to three times, depending on the need to clarify or verify data. The interviews were audiotaped and transcribed verbatim before data analysis was performed. Field notes were recorded to capture non-verbal information and note the researcher’s reflective processes and the participants were encouraged to speak freely and recount their personal experiences of diabetes. The interview questions were broad and open-ended, designed initially to identify concepts. Data collection continued until saturation was reached. Saturation occurred when no new codes or categories emerged from the analysis of data [23]. 

Analysis of the transcripts was guided by constant comparative analysis [25]. All audiotapes were transcribed verbatim immediately after each interview. Each transcript was read three times to enable the researcher to become familiar with the data. The transcript was then re-examined and read line by line by the first researcher to identify key codes. The process began with open coding. Coding, as used here, was a way of identifying and conceptualizing events, actions and meaning. In axial coding, the aim was to clarify how the subcategories that emerged were related to preliminary categories and then to compare how the preliminary categories were integrated with each other. Finally, the researchers focused on the meaningfulness and social interactions of a higher order to find a pattern based on social actions. ‘Memo – writing’ was used; Memos, as theoretical reflections and ideas based on data, were written down after every interview, during the analysis, and written in a freeway [25].

To ensure data trustworthiness, quality control was used to examine the fit of the findings. In addition to the debate, exchange of views and consensus with consultants and supervisors in various stages of the study, the used methodology, objective of the study, and the findings were presented to a fellow researcher familiar with qualitative research to confirm the logical procedure [25]. In cases where the findings were not confirmed, the concepts and the findings were reviewed and presented to the fellow researcher. Moreover, the long-term professional experience of the author with the considered phenomenon and the patients with diabetes was helpful.

In this study, after obtaining authorization from the Research Ethics Committee of Shahid Beheshti University of Medical Sciences, the study began. Written informed consent for participating in the study and recording interviews was obtained from the subjects. The place and time of the interviews were coordinated with the participant, and they were explained that the lack of participation had no effect on the medical services provided at the same institution. Participants had the right to withdraw from the study or deny responding to any question to which they were not willing to respond. Participants were assured that their information would remain confidential and anonymous, and they could have the results if they wished.

## Results

The study was based on data from 9 men and 19 women with type 2 diabetes from different urban and rural regions of Iran, between the ages of 36 and 72, educational levels from illiteracy up to Ph.D., and duration of diabetes ranged between 2 and 30 years.

The main category that indicated a challenge in treatment adherence in people with type 2 diabetes was “loss of time”. The factors that create this category and appear to be specific to Iran are: cultural habits and values, specific beliefs (believing diabetes was God’s will), age requirements (resistance to change), non-shared decision making, job conditions (a disproportionate treatment regimen with job conditions), and the reaction of others. 

In general, the factors influencing the process of adherence to treatment in the experiences of patients in the cultural and social context of Iran were classified into three main categories:

• Ineffective healthcare systems (non-shared decision making);
• Social pressures (job conditions, lack of harmony in the family);
• Personal inabilities (cultural habits and values, specific beliefs, the effect of age).

Because of the chronic and silent nature of diabetes, in most people with diabetes, the diagnosis does not coincide with the time of getting sick, and diagnosis is usually delayed, and pathological changes have already occurred in the body. At the time of diagnosis, these people do not have much time to lose. However, people’s dependency on past incorrect habits, which are the potential causes of diabetes, leads to conflicts, inconsistency, and challenges in adherence to treatment. According to participant’s statements, lifelong habits cannot be changed within a day, and making extensive behavioral changes and adherence to a complex care regimen to control the disease requires a team effort from people with diabetes, their family and the health professionals. The poor performance of the medical team in convincing people with diabetes to participate in the care plan has a negative effect on the adherence to treatment. A 48-year-old man with diabetes said: “It’s too late. We received the information when it was too late for us. We should have known the consequences from the beginning” said patient (P) 7. A 52-year-old man with diabetes said: “I have experienced everything ... It was my right to know everything about my conditions from the beginning, so I could decide for my health with their help... now it’s too late, does that heart come back? (P8)”.

### Ineffective healthcare systems

People with diabetes reported that they often received incomplete and sometimes contradictory information from healthcare providers; this information caused confusion. The lack of clear guidelines is a barrier to proper self-care and adherence to treatment for people with diabetes with low health literacy who are unable to find information and recognize correct information. A 36-year-old woman with diabetes said: “several doctors and nurses differently explained to me what to do and how to act in different conditions as if everyone had his theory about diabetes. Well, I do not know exactly what to do (P12)”. A 44-year-old woman with diabetes said: “Everyone has something to say; for example, the dietitian says you can eat these things, but my doctor said these are not good; I do not know what to do. I am afraid my condition will get worse (P10)”.

According to the participants report, having someone who knows and directs them to understand the health requirements and care plan easily is a good way to adhere to the treatment. A 49-year-old woman with diabetes said: “Usually, no one responds in hospitals. They pass the patients to each other or respond with short and incomplete sentences. Our problems remain the same, and we cannot solve them... (P16) “. A 54-year-old woman with diabetes said: “In the hospital, no one has the patience or time to see what problems we have or do not know... during my last day in the hospital, one of the inexperienced nurses came into the room, read a sheet and went away as if she did not know how to explain; in response to my questions, she said to ask the doctor (P5)”.

### Non-shared decision making

The prevailing idea of the treatment team (deciding for the patients rather than with the patients because they see the patients unable to make decisions in critical conditions) limits patient participation in the care plan, discouraging self-care. A 46-year-old woman with diabetes demanded more interaction between the patient and treatment team: “we want to talk with the doctor about things which are hard for us and find a solution. It is very difficult for many patients to do whatever the doctor says... (P3)”. A 58-year-old man with diabetes said: “the doctor does what he wishes; he does not care what I think... he does not ask me any questions whether I follow my diet or if I am exercising or taking my medicine... he just prescribes some medicine. At first, I do not know what they want to do, so I do not take the drugs properly ... doctors think that only they know everything. However, if they explain it to us, we would understand... (P11) “. 

### Cultural habits and values

An unhealthy lifestyle and dependence on bad habits lead to neglecting health behaviors. A 52-year old man said: ‘I used to eat rice with any food. Usually, our foods are fried, we do not eat many vegetables… although I know these things may be harmful to me, I cannot follow a diet plan (P2)’. Moreover, a person with diabetes is trapped in unrealistic beliefs regarding diabetes. These beliefs, which depend on family and social backgrounds, make it difficult for patients to adhere to treatment. For example, a 44-year old woman said: ‘My doctor said I have to inject insulin, but I will not accept it as long as possible. Insulin injection is a nightmare for me. I feel it is the end stage of the disease, I feel disappointed (P10)’. 

### Religious beliefs

Some participants’ religious beliefs, especially their belief in God’s will in getting sick, were closely related to their self-care behaviors. They believed that the disease is determined by God, and the end of the disease is in the hands of God; believing in this divine determinism prevents them from trying to recover or practice self-care. A 67-year-old woman said: ‘My destiny is to get diabetes… after this, whatever God wants, it will happen (P20)’.

A 72-year-old man said: ‘It is God’s will that I get diabetes. I’m satisfied with God’s pleasure... I just believe in God that I will not be worse (P17)’.

### Effect of Age

Change in lifestyle and everyday life is something more challenging than it seems. Change is not just taking medications; diet and regular control of blood sugar is the most difficult aspect of diabetes control. For this self-care, people require physical and psychological ability, motivation, and life expectancy. Often, middle-aged people need the help of others because of their low motivation, reduced life expectancy, or reduced physical abilities. For example, a 63-year-old woman with diabetes said: “I cannot do all that they say to do, to check my ulcers in the foot, to walk, to maintain insulin levels, to eat my food, how to eat, what to eat, to check my sugar level several times a day, to take tests, to visit my doctor... therefore, one of my children has to help me... I do not have the ability... I tell myself, what is the point of all these? I’m going to die in the end. My children remind me, they insist (P9)”. A 67-year-old man with diabetes said:” It’s so hard for us to lose weight … this is good for people who are young and healthy and want to live (P15)”.

### Job Conditions

People with high-stress levels who are busy are unable to manage their disease, diet and medication properly. Based on the fact that people have a part-time or full-time job, it is necessary to plan with the participation of the health professionals, the patient, and the family. A 47-year-old man with diabetes said: “When you work for 10 to 14 hours a day, it is hard to keep a diet and inject insulin twice a day. That is why I quit my treatment... (P21) “. A 46-year-old woman with diabetes said: “Because I work too much, I am constantly hungry and eat something... my stress level is high; I sometimes forget my diet and medications (P6)”.

### Reaction of Others

The inappropriate reaction of others was one of the barriers to regular adherence to treatment. Fear of judgment and commiseration of others, commiserating behavior of people, blaming the patient for the development of the disease lead to feelings of failure in the patient; subsequently, the patient may hide the disease in collective environments and even ignore the treatment regimen. A 56-year-old woman with diabetes said: “commiseration of people weakens morality of patients... that is why I do not say I am diabetic when I go parties ... I eat everything available at the party. I do not inject insulin at the parties (P3)”. A 68-year-old woman with diabetes said: “when I told my family that I have diabetes, they said that is my fault because I am fat ... this is painful ... So I do not tell anybody that I have diabetes, I do not even take medications in front of other people... (P19) “.

### Harmony in the family 

People with diabetes seek the support of their families; they noted that they are not able to change their lifestyle and diet without the cooperation of their family members. A majority of participants claimed that family members’ support and coordination, particularly spouses, facilitate adherence to treatment. For example, a 39-year-old man with diabetes said: “the family should be in harmony; they should reach an agreement on their behaviors and diet; this is very important. Otherwise, the patient will become indifferent soon (P14)”. A 56-year-old man with diabetes said: “my wife has long refused me to eat other food except for my diet. At home or out of the house, she eats her food without salt or fat, does not eat desserts and sweets that I do not eat. In addition, she walks every evening with me (P1)”. 

## Discussion

Adherence to treatment in Iranian people with diabetes has a context base (cultural habit, health beliefs, religious beliefs) and is an interactive process. Based on the results of this research, people with diabetes do not take their disease serious when encountering with it due to lack of serious health problems. This is due to the chronic nature of diabetes, so that many of people with diabetes are not aware of the serious side effects of the disease. For this reason, they are doubtful in accepting the treatment and changing their lifestyle. However, Torresan et al. showed that patients suffering from other chronic diseases, such as patients suffering from heart failure or cancer, are taking drugs regularly and adhere to treatment without question and doubt due to nature of the disease and symptoms such as pain [26]. The patients doubt of being sick and trying to challenge the disease occurs as a natural reaction in people with type 2 diabetes; as long as the patient has an opportunity to think about the treatment, some patients may spend much in this stage, losing the opportunity of preventing side effects and problems associated with the disease. Thus, early training of diabetic people can be useful in understanding the risks of the disease and having a fast response in front of the disease, changing their lifestyle. Also, Karimian et al. believed that it is recommended to strengthen discharge instructions given by nurses [27]. 

Beliefs about diabetes often relate to social understanding and people’s culture, which can affect adherence to treatment [28]. Self-management of the diet in diabetes requires the motivation and understanding of quantities and nutritional values. This knowledge and motivation in Iranian people with diabetes are influenced by social and cultural habits and they must understand that traditional, high-calorie foods, fried food and rice are harmful to them. This finding was confirmed by other studies [29, 30]. Therefore, there is a need to provide a more appropriate culture for care and attitude to these people with a greater focus. Also, the participants believed that God’s will would prevail because God predetermined the events, including the diagnosis of diabetes. The belief in predetermination, according to God’s will, could be an obstacle to disease control and self-care. In comparison with other studies, this is a new finding of the present study. Other studies showed that religious beliefs strengthen patients to endure diabetes [31, 32].

Also, in this study, participation in the decision-making of both the treatment team and patients regarding the care plan was very important in motivation and self-care of patients. This study showed that Iranian health professionals are unable to facilitate adherence to treatment. They insist on providing clinical care and treatment rather than education and counseling; they do not have enough time to listen to patients and train the patients and their families, while patients demand coordinated care models or healthcare interactions, which are accompanied by discussion and understanding. The patients prefer clinical decisions that are made collaboratively according to individual preferences of patients with alternative treatment options. Lewis and Newell also claimed that the lack of adequate information and quality care as barriers to continuity of care is a common phenomenon in developing countries [33]. Moreover, the study of Karimian et al. indicated a low quality of education in Iranian Hospitals [27]. Parchman et al. also considered patient-centeredness and balanced participation of the patient and medical team in planning care and treatment as activators of self-care, which is associated with adherence to treatment [34]. In a qualitative study by Booth et al., the patients and the medical team repeatedly stressed the importance of sufficient time for consultation with the patient and responding to their questions, giving information, and agreement on a course of action in accordance with patient’s needs [35] which can serve as a core of good quality care and a factor for empowering self-care behaviors [36, 37]. The increased number of nurses, educators and consultants, as well as adequate training plans, seems helpful in supporting self-efficacy of patients in the adherence to treatment. Moreover, a patient-centered approach of healthcare providers is effective in protecting the patients and eliminating the barriers.

This study showed that family members play an important role in improvement of treatment adherence; when a patient follows a healthy diet alone, and eats a separate meal at home, over time, he or she becomes tired and tends to focus on the family’s main meal. Same regarding physical activity; if done alone, it is very different from when all family members follow that lifestyle. In this regard, Atashzadeh-Shoorideh et al. also believe that the family plays a vital role in the promotion of patients’ self-efficacy and lifestyle improvement [38]. Therefore, according to Miller and DiMatteo, the identification of caregivers in the family helps the treatment team encourage and train them to participate in the process of adherence to the treatment and control of the disease [39].

Another key finding perceived by the participants in their experiences of living with diabetes was the inability to control the condition due to problems such as the cost of treatment, other studies (40, 41) finding the complexity of diet and drug regimen, and lack of physical activity as a challenge in adherence to the treatment process. Health care providers can simplify the treatment regimen (for example, reduce the frequency of medication during the day), negotiate with the patient about treatment priorities, give appointment reminders through follow-up programs, design realistic goals with the patient in order to increase patient collaboration and reduce the experience of failure to help patients change and monitor their condition. Moreover, the referral of people with diabetes to associations or charities for financial and educational support and the introduction of able counterparts can be helpful in this regard.

Data of this study implies the need of patients for psychosocial support regarding the permanent adherence to treatment. Participants reported that inappropriate reactions, different and negative attitude of others to diabetes lead patients to ignore the diet in collective environments. This attitude and the prevailing view of society rooted in the heavy responsibility of the patient to take multiple aspects of diabetes management or involvement with uncontrolled side effects of the disease can even have a negative effect on jobs and income of patients. Chew et al. also noted the need of patients for psychological and social support through healthcare system and society for effective self-management of diabetes. They also considered the very important effects of support on emotions, perceptions and self-efficacy of patients. They emphasized that effective management of diabetes by the medical staff and the society needs psychology and recognition of the relationship between encouragement and hope and health-related behaviors [42]. Hence, healthcare providers and various media can effectively change this negative attitude.

## Conclusions

This study noted an important part of healthcare. Poor adherence to treatment is a major public health problem in most countries, especially developing countries like Iran. It can be helpful to understand why Iranian people with diabetes do not adhere to their treatment, how they think and feel about social and cultural contexts, how they can change their beliefs and attitudes, and act differently by developing thoughts and interacting with others. The findings of the present study showed that people with diabetes go through a trial and error in learning to achieve adherence to treatment, and, in this way, they need help and support to adherence and adaptation to treatment more quickly and without complications. It is certainly not easy to make large behavioral changes to achieve treatment compliance, and each new stage of treatment may be associated with reluctance and resistance to change. In this way, people need time to accept various medical and nutritional treatments and a new lifestyle. In fact, the time factor is a bridge between the first emotional response to disease and adaptation, or opposition and non-adaptation and challenge. If the care plan is designed to encourage the active participation of the person with diabetes, between the person and the treatment team in the shortest time, the time to achieve compliance is shorter and will bring the least side effects for the person. In fact, the support of the treatment team provides an opportunity for the person with diabetes to gain information, gain experience, and reach the area of adaptation to treatment and lifestyle changes more quickly and with the least complications. The findings of this study can be used to design comprehensive programs for empowering people with diabetes and promoting adherence to treatment. 

## Conflict of Interest

The authors declare that there is no conflict of interest.
